# Pre-Radiotherapy Progression after Surgery of Newly Diagnosed Glioblastoma: Corroboration of New Prognostic Variable

**DOI:** 10.3390/diagnostics10090676

**Published:** 2020-09-05

**Authors:** Radek Lakomy, Tomas Kazda, Iveta Selingerova, Alexandr Poprach, Petr Pospisil, Renata Belanova, Pavel Fadrus, Martin Smrcka, Vaclav Vybihal, Radim Jancalek, Igor Kiss, Katarina Muckova, Michal Hendrych, Andrea Knight, Jiri Sana, Pavel Slampa, Ondrej Slaby

**Affiliations:** 1 Department of Comprehensive Cancer Care, Masaryk Memorial Cancer Institute, 656 53 Brno, Czech Republic; lakomy@mou.cz (R.L.); poprach@mou.cz (A.P.); kiss@mou.cz (I.K.); jiri.sana@mou.cz (J.S.); 2 Department of Comprehensive Cancer Care, Faculty of Medicine, Masaryk University, 625 00 Brno, Czech Republic; 3 Department of Radiation Oncology, Masaryk Memorial Cancer Institute, 656 53 Brno, Czech Republic; ppospisil@mou.cz (P.P.); slampa@mou.cz (P.S.); 4 Department of Radiation Oncology, Faculty of Medicine, Masaryk University, 625 00 Brno, Czech Republic; 5 Central European Institute of Technology, Masaryk University, Kamenice 5, 625 00 Brno, Czech Republic; ondrej.slaby@ceitec.muni.cz; 6 Research Center for Applied Molecular Oncology, Masaryk Memorial Cancer Institute, 656 53 Brno, Czech Republic; iveta.selingerova@mou.cz; 7 Department of Radiology, Masaryk Memorial Cancer Institute, 656 53 Brno, Czech Republic; belanova@mou.cz; 8 Faculty of Medicine, Masaryk University, 625 00 Brno, Czech Republic; 9 Department of Neurosurgery, University Hospital Brno, and Faculty of Medicine, Masaryk University, 625 00 Brno, Czech Republic; fadrus.pavel@fnbrno.cz (P.F.); smrcka.martin@fnbrno.cz (M.S.); vybihal.vaclav@fnbrno.cz (V.V.); 10 Department of Neurosurgery, St. Anne’s University Hospital Brno, 656 91 Brno, Czech Republic; radim.jancalek@fnusa.cz; 11 Department of Neurosurgery, St. Anne’s University Hospital Brno, Faculty of Medicine, Masaryk University, 625 00 Brno, Czech Republic; 12 Department of Pathology, University Hospital Brno and Faculty of Medicine, Masaryk University, 625 00 Brno, Czech Republic; muckova.katarina@fnbrno.cz; 13 First Department of Pathology, St. Anne’s University Hospital and Faculty of Medicine, Masaryk University, 656 91 Brno, Czech Republic; michal.hendrych@fnusa.cz; 14 Department of Pathological Physiology, Faculty of Medicine, Gamma Delta T Cell Laboratory, Masaryk University, 625 00 Brno, Czech Republic; knight@med.muni.cz; 15 Department of Biology, Faculty of Medicine, Masaryk University, 625 00 Brno, Czech Republic

**Keywords:** glioblastoma, chemotherapy, radiotherapy, rapid early progression, overall survival

## Abstract

Background: The aim of this retrospective study is to assess the incidence, localization, and potential predictors of rapid early progression (REP) prior to initiation of radiotherapy in newly diagnosed glioblastoma patients and to compare survival outcomes in cohorts with or without REP in relation to the treatment. Methods: We assessed a consecutive cohort of 155 patients with histologically confirmed irradiated glioblastoma from 1/2014 to 12/2017. A total of 90 patients with preoperative, postoperative, and planning MRI were analyzed. Results: Median age 59 years, 59% men, and 39 patients (43%) underwent gross total tumor resection. The Stupp regimen was indicated to 64 patients (71%); 26 patients (29%) underwent radiotherapy alone. REP on planning MRI performed shortly prior to radiotherapy was found in 46 (51%) patients, most often within the surgical cavity wall, and the main predictor for REP was non-radical surgery (p < 0.001). The presence of REP was confirmed as a strong negative prognostic factor; median overall survival (OS) in patients with REP was 10.7 vs. 18.7 months and 2-year survival was 15.6% vs. 37.7% (hazard ratio HR 0.53 for those without REP; *p* = 0.007). Interestingly, the REP occurrence effect on survival outcome was significantly different in younger patients (≤ 50 years) and older patients (> 50 years) for OS (*p* = 0.047) and non-significantly for PFS (*p* = 0.341). In younger patients, REP was a stronger negative prognostic factor, probably due to more aggressive behavior. Patients with REP who were indicated for the Stupp regimen had longer OS compared to radiotherapy alone (median OS 16.0 vs 7.5; HR = 0.5, *p* = 0.022; 2-year survival 22.3% vs. 5.6%). The interval between surgery and the initiation of radiotherapy were not prognostic in either the entire cohort or in patients with REP. Conclusion: Especially in the subgroup of patients without radical resection, one may recommend as early initiation of radiotherapy as possible. The phenomenon of REP should be recognized as an integral part of stratification factors in future prospective clinical trials enrolling patients before initiation of radiotherapy.

## 1. Introduction

The Stupp regimen is still the standard of care for patients with newly diagnosed glioblastoma, with only a few reports indicating possible improvement in the past decade [1,2]. Apart from the possible role of tumor treating fields [3], the update in treatment guidelines was mainly related to modifications of already known procedures (abbreviated chemoradiotherapy, or combination of temozolomide and lomustine) [4,5]. Prognostic and predictive biomarkers guide the indication for optimal treatment. Besides classical clinical prognosticators, biomarkers such as promoter methylation of the O6-methylguanine-DNA-methyltransferase (MGMT) gene or isocitrate dehydrogenase (IDH) 1 and 2 mutations moved into daily practice and became an integral part of diagnosis [6,7,8,9]. With the long-lasting lack of new effective therapeutics, further biomarkers for a suitable indication of the currently used modifications of temozolomide-based chemoradiotherapy are one way to improve the care of these patients.

The phenomenon of postoperative rapid early progression (REP) has only recently been explored with increasingly available magnetic resonance imaging (MRI) for both postsurgery and pre-radiotherapy (pre-RT) indication and is currently of high interest. REP diagnosis is based on a comparison of early postoperative MRI findings (up to 72 h postoperatively) and planning pre-RT MRI. Only a few studies retrospectively evaluated REP and indicated almost up to 50% risk of development of REP, regardless of the waiting time until the start of radiotherapy (RT) [10,11,12]. Clearly, these patients biased previous clinical trials, where no routine pre-RT MRI examination was performed. Currently, the treatment of these patients is not different from patients without REP, and if so, it is a purely individual approach.

The aim of this retrospective study is to evaluate the incidence and localization of REP in a consecutive cohort of patients treated, out of the frame of clinical trials (real-world evidence data). The aim is also to describe clinical factors associated with REP in glioblastoma and to describe the effect of REP and treatment on survival.

## 2. Materials and Methods 

### 2.1. Patients and Treatment

**Figure 1:**
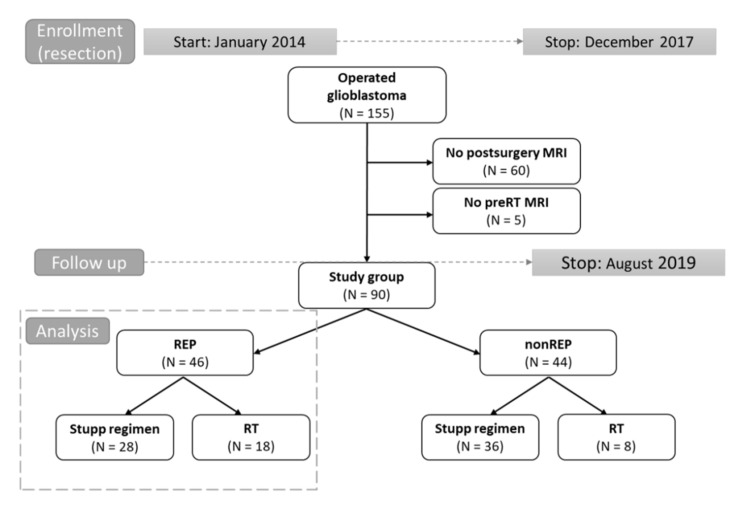
Design of study (flow chart). Grey dot line: time to study enrolment and follow up, respectively. Black line arrows: division into parts. Grey dot line box: cohort analyzed in more detailes.

A consecutive cohort of 155 histologically confirmed glioblastoma patients, who were indicated via a multidisciplinary neuro-oncology board to adjuvant or palliative radiotherapy between 01/2014 and 12/2017, were screened for eligibility to this retrospective study (Figure 1). All patients were treated outside of clinical trials. Those with available early postoperative MRI (up to 72 h) evaluating the extent of surgery and those who had also performed pre-RT MRI were eligible for assessment of REP. The subgroup of patients who were indicated for the treatment according to the Stupp regimen was further analyzed in more detail. All patients signed standard informed consent to treatment and consent to processing their data for scientific purposes in a pseudonymized form. The study was approved by Institutional Review Board No. 2020/1206/MOU, JID: 315 453, approved on day month year. Institutional Review Board No. 2020/1206/MOU, JID: 315 453, approved on 18 June 2019.

Clinical and imaging data were retrieved from electronic medical records for further statistical analysis. Radiotherapy was performed in all patients. Planning pre-RT MRI (including postcontrast T1 weighted scan with submillimeter slices) was rigidly registered to planning CT scan for proper RT target and organs-at-risk definition. Individual prescription of RT dose and scheduling was guided mainly by the patient’s performance status and by volume, size, shape, and location of the target volume. Both standard of care approaches in target volume definitions were employed in patients eligible for treatment by the Stupp regimen (60 Gy in 30 fractions), the Radiation Therapy Oncology Group (RTOG contouring approach) that defines two clinical target volumes accommodating hyperintensity at T2/FLAIR MRI in addition to T1 contrast-enhanced MRI [13], and the European Organization for Research and Treatment of Cancer (EORTC single-phase contouring approach) that defines one target utilizing mainly T1 post-contrast MRI [14]. In patients with REP, the single target EORTC approach was preferably performed. The RT plan was prepared employing the treatment planning system EclipseTM (Varian medical systems, Palo Alto, CA, USA) and delivered on linear accelerators Varian Clinac iX or TrueBeam (Varian medical systems, Palo Alto, CA, USA). Abbreviated RT courses (for example, 15 × 2.7 or 10 × 3.4 Gy) were indicated according to the treating physician, reflecting the individual patient’s performance status and disease.

Concurrent chemoradiotherapy and adjuvant chemotherapy were prescribed according to the original Stupp protocol [1]. Temozolomide (75 mg/m2) was administered on days 1 through 42 with concurrent RT (60 Gy). After 4 weeks, treatment was followed by the administration of temozolomide alone (150 to 200 mg/m2) on days 1–5 in six consecutive 4-week cycles or to progression. The prophylaxis against *Pneumocystis jirovecii* pneumonia was at the discretion of the treating physician. In patients with an abbreviated course of RT, concurrent chemotherapy was usually not indicated and was initiated after the end of RT based on the patient’s actual performance status. Treatment at progression was very individualized, with options for resurgery, reirradiation, temozolomide rechallenge, palliative chemotherapy (mostly lomustine), or symptomatic treatment.

### 2.2. Imaging Evaluation

All diagnostic MRIs were evaluated by two independent radiologists as part of the standard of care in our institution. In the case of discordance, patients were referred to the discussion on the neuro-oncology tumor board as well. Response to treatment was evaluated based on regular follow up MRI scanning. The first post (chemo) radiotherapy MRI was usually performed 4–6 weeks after the last RT session, followed by regular MRI every 3 months unless clinically indicated for earlier examination. No routine RANO criteria [15] were employed and MRI was visually evaluated by the servicing radiologist. Unclear findings (as was suspected pseudoprogression) were reviewed by a multidisciplinary neuro-oncology board, mostly with recommendations for earlier control exams with or without the change of treatment or with suggestions for advanced MRI methods [16]. 

The pre-RT MRI was retrospectively evaluated by an experienced radiation oncologist (TK) and doublechecked by a neuroradiologist (RB). Progression already presented on planning MRI was considered only in patients who had available early postsurgery (within 72 h) control MRI enabling a clear definition of eventual postsurgery residual disease. Criteria for REP were as follows: (1) increase in postsurgery residual disease (T1 weighted post contrast MRI) for ≥25% in any dimension; (2) occurrence of a new enhancing lesion; (3) unambiguous progression of enhancing lesion (in multifocal glioblastomas where only some nodules were amenable to surgery). The localization of REP was categorized as follows: (1) progression of postsurgery residuum; (2) new enhancing satellite; (3) new enhancement in the wall of resection cavity; or (4) progression of tumor which was not operated on in patients with multicentric tumors. 

### 2.3. Statistical Analysis

Patient and treatment characteristics were described using standard summary statistics, i.e., median and interquartile range (IQR) for continuous variables and frequency distributions for categorical variables. The comparison of these characteristics in patients with and without the occurrence of REP was performed using Fisher’s exact test, a chi-squared test, or a Mann–Whitney test, as appropriate. Overall survival (OS) and progression-free survival (PFS) were considered as survival outcome. OS was defined as the time from the date of neurosurgery resection to the date of death from tumor cause. PFS was defined as the time from the date of initiation of RT until progression or death from tumor cause. Survival probabilities were calculated by the Kaplan–Meier method. Survival curves were compared using the log-rank test. The Cox proportional hazard model was used to perform the univariable and multivariable analysis. The proportional hazard assumption was verified based on scaled Schoenfeld residuals. Stepwise backward selection was performed to obtain characteristics independently associated with OS and PFS. Stratified models were used for the assessment of the effect of treatment or age in patients with and without the occurrence of REP. All statistical analyses were performed employing R version 4.0.0 [17], and the significance level of 0.05 was considered.

## 3. Results

### 3.1. Patients Characteristics

A total of 155 patients who were indicated for postoperative oncology treatment were screened for eligibility, and 90/155 (58%) met the inclusion criteria and had undergone both postsurgery as well as pre-RT MRI (Figure 1). The median age was 59 years, with 23% of patients being younger than 50 years. Gross total resection (GTR) was achieved in 39/90 (43%) patients, and 34/90 (38%) were in excellent overall performance status with Eastern Cooperative Oncology Group (ECOG) status 0. MGMT methylation was present in 26% and IDH mutation in 8% of patients (total of 53 evaluated patients). The subgroup of 64 patients (64/90; 71%) was indicated for concurrent chemoradiotherapy and was further analyzed in detail. The other patients’ diagnostic and treatment characteristics are summarized in Table 1; Table 2.

### 3.2. Rapid Early Progression

REP was presented in 46 out of 90 evaluated patients (51%). In the majority of patients, REP was presented as a progression of postsurgery residuum (31/46; 67%) or as a new enhancement in the wall of the resection cavity (22/46; 48%). Only 6/46 (13%) REP presented by a new enhancing lesion and 10/46 (22%) by the progression of the tumor, which was not operated on in patients with multicentric tumors. The occurrence of REP was significantly associated with the extent of resection (78% of patients with REP vs. 34% of patients without REP, after non-radical resection; *p* < 0.001). The other evaluated pre-RT diagnostic variables (age, sex, performance status, etc.) were not significantly associated with the development of REP (Table 1).

**Figure 2:**
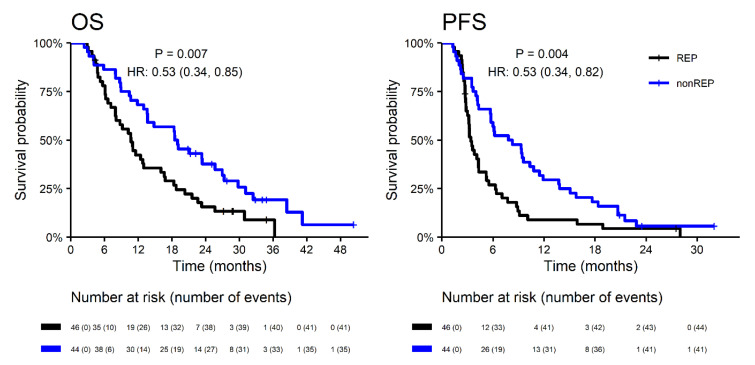
Overall survival and progression-free survival in patients with REP vs. non-REP. OS—overall survival; PFS—progression free survival; HR—hazard ration; REP—rapid early progression.

**Figure 3:**
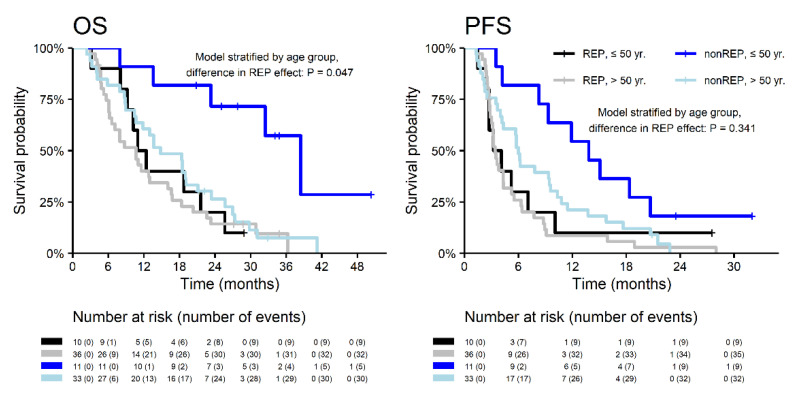
Overall survival and progression-free survival in patients with REP and non-REP in relation to age.

With a median follow up (measured from neurosurgery resection) of 34.1 months, the median OS was significantly longer in patients without REP (18.7 vs. 10.7 months; HR 0.53; *p* = 0.007) with corresponding 2-year survival 37.7% vs. 15.6%. A similar effect was observed for PFS (Figure 2). Interestingly, the REP occurrence effect on survival outcome is significantly different in younger patients (≤50 years) and older patients (>50 years) for OS (*p* = 0.047) and non-significantly for PFS (*p* = 0.341). In younger patients (≤50 years), REP occurrence is a negative prognostic factor, probably in relation to more aggressive glioblastoma behavior at a younger age (Figure 3).

**Figure 4:**
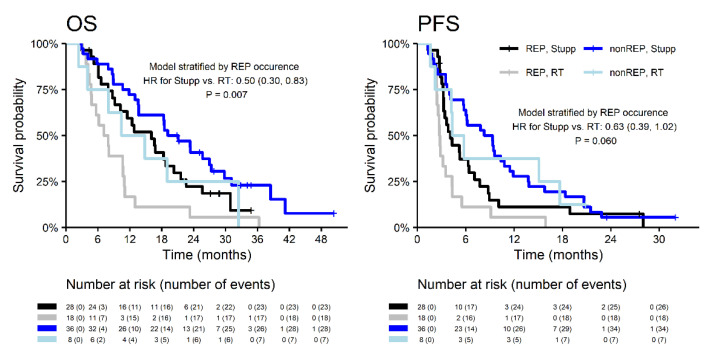
Survival outcomes of patients with REP and non-REP in relation to the treatment.

Indication to concurrent chemoradiotherapy (the Stupp regimen) was more common in the subgroup without REP (82% vs. 61%; *p* = 0.037), as was summarized in Table 2. OS and PFS were significantly better in patients indicated for the Stupp regimen in both subgroups with and without REP (Figure 4). The median OS of patients with REP who were indicated for the Stupp regimen was 16.0 (2-year OS 22.3%). The median OS of patients treated by RT alone was 7.5 months (2-year OS 5.6%) (Table 3). The model stratified by REP showed a 50% lower risk of death, and a 37% lower risk of progression in patients indicated concurrent chemoradiotherapy (OS: HR = 0.5, *p* = 0.007; PFS: HR = 0.63, *p* = 0.060).

**Figure 5:**
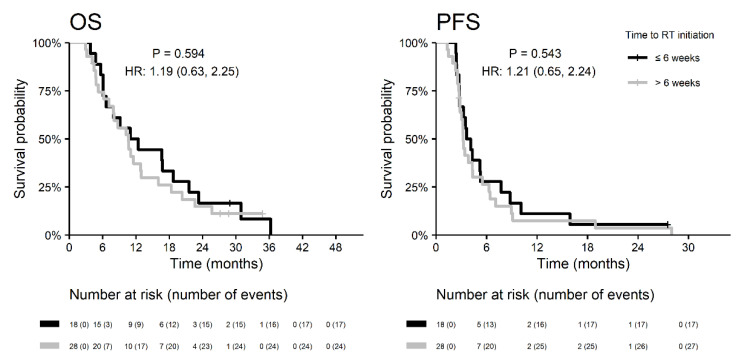
Survival outcomes of patients with REP in relation to the start of radiotherapy.

The median time to initiation of radiotherapy was 6.7 weeks and was similar in both groups of patients (6.6 vs. 6.8 weeks in patients with and without REP, respectively). In the REP subgroup, both OS and PFS were similar in patients undergoing RT within six weeks after resection as in patients with a longer initiation time (Figure 5). Target definition for radiotherapy planning according to EORTC (the one same target for the whole course of RT) was more commonly employed in the subgroup of patients with REP (65%) vs. in patients without REP (36%; *p* = 0.011). Nevertheless, the OS of patients with REP did not differ with respect to contouring strategy (HR 0.9 for RTOG vs. EORTC; *p* = 0.824).

**Figure 6:**
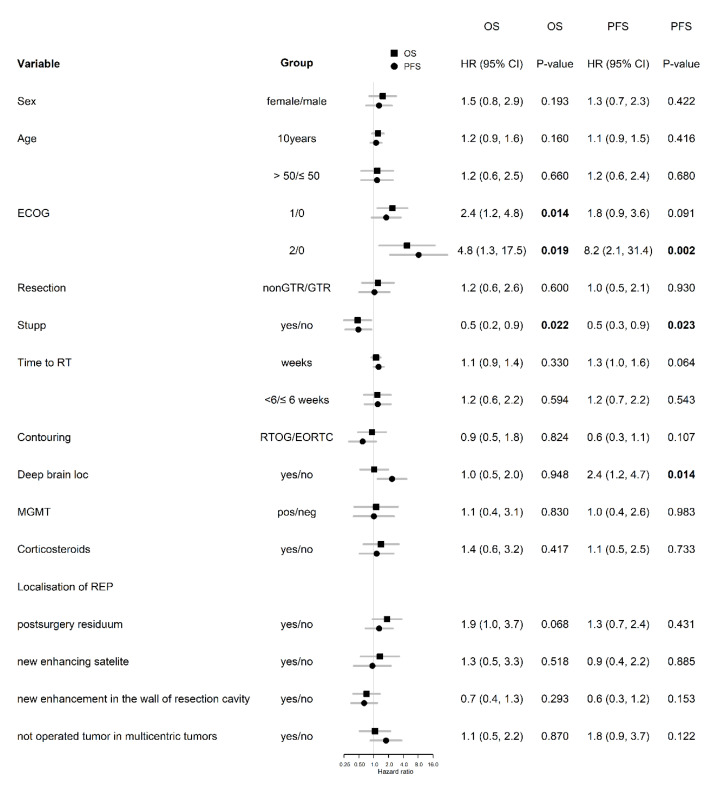
Univariable analysis in patients with REP. Number in bold are < 0.05.

Based on univariable analysis of 46 patients with REP, the lower overall performance status (the median OS 16.8 vs. 11.0 vs. 5.8 months in patients with ECOG 0 vs. 1 vs. 2; *p* = 0.011), and indication to concurrent chemoradiotherapy (HR 0.50; *p* = 0.022 for OS) was positively associated with OS and PFS (Figure 6). REP presented as a progression of postsurgery residuum was a negative prognostic factor of OS with the borderline level of statistical significance (HR 1.9; *p* = 0.068). Deep brain tumor location was a significant negative prognostic factor for PFS (HR 2.4; *p* = 0.014), but not for OS (HR 1.0; *p* = 0.948). The other prognostic variables (age, sex, the extent of resection, MGMT status, the location of REP) were not significant in the univariable analysis (Figure 6). IDH mutation was not evaluated in a univariable analysis due to low numbers of positive patients. According to the multivariable analysis of patients with REP (Table 4), the extent of resection and Stupp regimen are independently associated with OS, and performance status and deep brain tumor location are independently associated with PFS. 

## 4. Discussion

A high proportion of glioblastoma patients indicated for adjuvant oncology treatment developed rapid early progression in this retrospective analysis of an unselected cohort of consecutive patients treated outside of clinical trials. About half of the patients (46/90; 51%) progressed between surgery and initiation of adjuvant RT, regardless of waiting time to RT initiation. High incidence of REP and reports of overall survival are in accordance with other retrospective published studies [10,11,12]. The only one clinical negative predictive factor for the development of REP in our cohort was non-radical surgery, confirming the overall prognostic value of surgical radicality in glioblastoma [1]. Further studies evaluating potential biomarkers of REP are highly warranted.

The question of optimal timing of RT initiation, the first logical argument for the risk of REP in a specific patient, is still unanswered. Published studies that evaluated this issue are inconclusive with different waiting times, ranging from 37 to 56 days after surgery [18,19,20,21,22,23]. Some reported no effect of waiting time on the OS. In the broad analysis of 2855 patients enrolled in 16 RTOG trials, Blumenthal et al. described even better outcomes in patients with the mild postponement of RT (4–6 weeks) comparing to early initiation of RT within 2 weeks after surgery [24]. One may assume the need for recovery from secondary edema and hypoxia to be a prerequisite for RT effect on radioresistant glioblastoma. However, considering glioblastoma aggressivity with doubling time reported about 24 days, it is recommended to avoid unnecessary delay in RT initiation [25,26,27,28]. 

Development of REP represents an important, and not yet described in detail, negative prognostic factor (median OS 10.7 vs. 18.7 months in our cohort). We confirmed other well-known prognostic factors, such as performance status and the ability to undergo the Stupp regimen. The question of eventual administration of chemotherapy for over 6 months remains to be answered. As expected, worse OS was in the subgroup of patients with REP who were treated by RT alone (OS 7.5 months).

The majority of patients with REP develop central progression within the initial lesion of the cavity. Modification of RT targeting and techniques including employment of planning PET may be another way how to improve the outcomes of this unfavorable group of patients [29,30]. Precise knowledge of tumor biology may also add to the guidance of optimal treatment (prediction for more invasive forms of glioblastoma and risk of distant satellites). MGMT promoter methylation is both a prognostic and predictive marker in an REP group of patients, as described by Palmer et al.: patients with both REP and MGMT methylation reached significantly longer survival compared to those with REP and MGMT intact (16.5 vs. 10.2 months, *p* = 0.033) [12]. In our cohort, we did not observe any role of MGMT (*p* = 0.830). However, only 23/46 (50%) patients with REP were examined, which could significantly affect our analysis. Palmer’s study evaluated MGMT promoter methylation, however, there may be many different genetic mutations and molecular characteristics specific to a subset of patients that predispose to REP and poor treatment response. It can be assumed that other important molecular markers such as IDH and pTERT (Telomerase reverse transcriptase gene promoter) also influence the prognosis and rapid progression in patients with glioblastomas [31,32,33].

In our clinical practice, MGMT is more likely to be investigated in elderly patients and in patients unable to undergo intensive postoperative treatment. For all others, we indicate the Stupp regimen regardless of MGMT methylation. It may be hypothesized that tumors with REP represent more aggressive disease, which may be associated with higher tumor mutation burden and neoantigens, relevant biomarkers for immunotherapy. On the other hand, unlike in other tumors, immunotherapy in glioblastoma did not prove clear effectivity so far, including immune checkpoint inhibitors or dendritic cell vaccines [34,35,36]. Analysis of REP patients may provide new insights into the biology of this aggressive tumor and potentially reveal new targets for cancer therapy.

An inherent limitation of our study is its retrospective nature, related also to limited possibility for molecular analyses. Ongoing work may provide more information, especially with the analysis of molecular biomarkers of REP. In future prospective studies, advanced MRI techniques such as MRI spectroscopy or diffusion-weighted MRI may play a role in the differential diagnosis of REP and postoperative changes, as does ischemia, for example. 

## 5. Conclusions

The extent of surgery remains one of the most important prognostic factors in glioblastoma, affecting not only general OS but also the risk of REP development. Especially in the subgroup of patients without radical resection, one may recommend as early initiation of radiotherapy as possible. The phenomenon of REP should be recognized as an integral part of stratification factors in future prospective clinical trials enrolling patients before the initiation of RT. 

## Figures and Tables

**Table 1:** Basic patients’ characteristics of cohort with REP and non-REP.

Study Cohort (*n* = 90)	All	REP	Non-REP	*p*-Value
No. of patients	90 (100%)	46 (51%)	44 (49%)	
**Age (years)**				
median (IQR)	59.3 (51.1, 65.2)	60.0 (52.2, 67.8)	57.1 (50.6, 63.5)	*p* = 0.180
≤50	21 (23%)	10 (22%)	11 (25%)	*p* = 0.805
**Men**	53 (59%)	27 (59%)	26 (59)%	*p* > 0.999
**Performance status (ECOG) and Karnofsky index**				
ECOG 0 (KI 90–100%)	34 (38%)	17 (37%)	17 (39%)	*p* = 0.868
ECOG 1 (KI 70–80%)	49 (54%)	26 (57%)	23 (52%)	
ECOG 2 (KI 50–60%)	7 (8%)	3 (7%)	4 (9%)	
**Tumor location**				
deep brain location	21 (23%)	14 (30%)	7 (16%)	*p* = 0.136
**Extent of resection**				
GTR	39 (43%)	10 (22%)	29 (66%)	*p* < 0.001
STR	44 (49%)	31 (67%)	13 (30%)	
Partial resection or biopsy	7 (8%)	5 (11%)	2 (5%)	
**Extent of resection**				
GTR	39 (43%)	10 (22%)	29 (66%)	*p* < 0.001
Non-GTR	51 (57%)	36 (78%)	15 (34%)	
**IDH status**				
Mutated/evaluated	4/53 (8%)	1/24 (4%)	3/29 (10%)	
**MGMT status**				
Methylated/evaluated	14/53 (26%)	6/23 (26%)	8/30 (27%)	*p* > 0.999
**Localization of REP**				
Postsurgery residuum		31/46 (67%)		
New enhancing satellite		6/46 (13%)		
New enhancement in the wall of resection cavity		22/46 (48%)		
Not operated tumor in multicentric tumors		10/46 (22%)		

Abbreviations: REP—rapid early progression; ECOG—Eastern Cooperative Oncology Group; GTR—gross total resection; non-GTR—non gross total resection; STR—subtotal resection; MGMT—O6-methylguanine-DNA-methyltransferase; IDH—Isocitrate dehydrogenase; IQR—interquartile ratio.

**Table 2:** Patients’ treatment.

Study Cohort (*n* = 90)	All (*n* = 90)	REP (*n* = 46)	Non-REP (*n* = 44)	*p*-Value
**Time to RT initiation**				
Median (weeks; IQR)	6.7 (5.9, 7.3)	6.6 (5.9 7.1)	6.8 (5.8, 7.5)	*p* = 0.981
>6 weeks	56 (62%)	28 (61%)	28 (64%)	*p* = 0.830
**Radiotherapy**				
RT technique IMRT	89 (99%)	46 (100%)	43 (98%)	
RT technique other	1 (1%)	0 (0)	1 (2%)	
median dose (Gy; IQR)	60 (50, 60)	60 (43, 60)	60 (60, 60)	*p* = 0.024
pts. receiving ≥ 90% of prescribed dose	82 (91%)	43 (93%)	39 (89%)	*p* = 0.480
contouring approach EORTC	46 (51%)	30 (65%)	16 (36%)	*p* = 0.011
contouring approach RTOG	43 (48%)	16 (35%)	27 (62%)	
contouring unknown	1/90 (1%)	0/46 (0)	1/44 (2%)	
**Chemoradiotherapy (Stupp regimen)**				
No. of patients	64 (71%)	28 (61%)	36 (82%)	*p* = 0.037
median (days; IQR)	42 (30, 45)	41.5 (23, 43)	43 (39, 46)	*p* = 0.095
corticosteroids use	62 (69%)	35 (76%)	27 (61%)	*p* = 0.151
**Adjuvant chemotherapy**				
No. of patients	43 (48%)	16 (35%)	27 (61%)	*p* = 0.020
No. of cycles: median (IQR)	4.5 (2, 6)	3.5 (1, 6)	5 (3, 6)	*p* = 0.242
No. of cycles: ≥ 3	32/43 (74%)	8/16 (50%)	24/27 (89%)	*p* = 0.016
No. of cycles: ≥ 6	21/43 (49%)	7/16 (44%)	14/27 (52%)	*p* = 0.761
**Treatment after progression**				
No. of patients	42	22	20	*p* > 0.999
surgery	7 (17%)	4 (18%)	3 (15%)	
surgery + chemoradiotherapy	1 (2%)	0 (0)	1 (5%)	
surgery + chemotherapy	8 (19%)	2 (9%)	6 (30%)	
chemotherapy	18 (43%)	13 (59%)	5 (25%)	
reirradiation	6 (14%)	2 (9%)	4 (20%)	
reirradiation + chemotherapy	2 (5%)	1 (5%)	1 (5%)	

Abbreviations: IQR—interquartile ratio; IMRT—intensity modulated radiotherapy; EORTC—European Organization for Research and Treatment of Cancer; RTOG—Radiation Therapy Oncology Group; GBM—glioblastoma; CHT/RT—chemoradiotherapy; CHT—chemotherapy; RT—radiotherapy.

**Table 3:** Survival outcomes in patients with REP and non-REP in relation to the treatment.

	REP (*n* = 46)		Non-REP (*n* = 44)	
	Median follow up31.9 (28.7, NA)		Median follow up 34.1 (32.9, NA)	
	Stupp regimen (*n* = 28)	RT (*n* = 18)	Stupp regimen (*n* = 36)	RT (*n* = 8)
**Overall survival**				
Median (months)	16.0 (10.2, 21.6)	7.5 (4.8, 11.0)	20.1 (13.6, 29.8)	12.6 (8.0, NA)
1-year	59.3 (43.4, 81.1)	16.7 (5.9, 46.8)	72.2 (59.0, 88.4)	50.0 (25.0, 100.0)
2-year	22.3 (11.0, 45.1)	5.6 (0.8, 37.3)	40.8 (27.3, 60.9)	25.0 (7.5, 83.0)
3-year	9.3 (1.9, 45.7)	5.6 (0.8, 37.3)	22.9 (11.9, 44.1)	0.0 (NA, NA)
**Progression-free survival**				
Median (months)	4.1 (3.2, 7.1)	2.8 (2.4, 4.3)	8.8 (5.8, 11.5)	5.0 (4.2, NA)
1-year	11.2 (3.8, 32.4)	5.6 (0.8, 37.3)	27.8 (16.4, 47.0)	37.5 (15.3, 91.7)
2-year	7.4 (2.0, 28.2)	0.0 (NA, NA)	5.6 (1.4, 21.4)	12.5 (2.0, 78.2)

Abbreviations: REP—rapid early progression; Stupp regimen—concomitant chemoradiotherapy and adjuvant chemotherapy with temozolomide; RT—radiotherapy; NA—Not Available.

**Table 4:** Multivariable analysis in patients with REP.

		OS		PFS	
		HR (95% CI)	*p*-Value	HR (95% CI)	*p*-Value
**Performance status (ECOG)**	**1/0**			2.3 (1.1,4.5)	0.033
	**2/0**			16.6 (3.9,70)	<0.001
**Extent of resection**	**non-GTR/GTR**	2.2 (0.9,5.2)	0.088		
**Stupp regimen**	**yes/no**	0.3 (0.1,0.7)	0.003		
**deep brain location**	**yes/no**			3.1 (1.5,6.7)	0.003

Abbreviations: OS—overall survival; HR—hazard ratio; CI—confidence interval; PFS—progression free survival; ECOG—Eastern Cooperative Oncology Group; GTR—Gross total resection.
